# Group-based variant calling leveraging next-generation supercomputing for large-scale whole-genome sequencing studies

**DOI:** 10.1186/s12859-015-0736-4

**Published:** 2015-09-22

**Authors:** Kristopher A. Standish, Tristan M. Carland, Glenn K. Lockwood, Wayne Pfeiffer, Mahidhar Tatineni, C Chris Huang, Sarah Lamberth, Yauheniya Cherkas, Carrie Brodmerkel, Ed Jaeger, Lance Smith, Gunaretnam Rajagopal, Mark E. Curran, Nicholas J. Schork

**Affiliations:** Biomedical Sciences Graduate Program, University of California, San Diego, Gilman Drive, La Jolla, 92092 CA USA; Human Biology, J. Craig Venter Institute, 4120 Capricorn Lane, La Jolla, 92092 CA USA; San Diego Supercomputer Center, University of California, San Diego, Gilman Drive, La Jolla, 92092 CA USA; Systems Pharmacology & Biomarkers (Immunology), Janssen R&D LLC, Springhouse, PA USA; R&D IT, Janssen R&D LLC, Springhouse, PA USA

**Keywords:** Variant calling, Supercomputing, Whole-genome sequencing

## Abstract

**Motivation:**

Next-generation sequencing (NGS) technologies have become much more efficient, allowing whole human genomes to be sequenced faster and cheaper than ever before. However, processing the raw sequence reads associated with NGS technologies requires care and sophistication in order to draw compelling inferences about phenotypic consequences of variation in human genomes. It has been shown that different approaches to variant calling from NGS data can lead to different conclusions. Ensuring appropriate accuracy and quality in variant calling can come at a computational cost.

**Results:**

We describe our experience implementing and evaluating a group-based approach to calling variants on large numbers of whole human genomes. We explore the influence of many factors that may impact the accuracy and efficiency of group-based variant calling, including group size, the biogeographical backgrounds of the individuals who have been sequenced, and the computing environment used. We make efficient use of the Gordon supercomputer cluster at the San Diego Supercomputer Center by incorporating job-packing and parallelization considerations into our workflow while calling variants on 437 whole human genomes generated as part of large association study.

**Conclusions:**

We ultimately find that our workflow resulted in high-quality variant calls in a computationally efficient manner. We argue that studies like ours should motivate further investigations combining hardware-oriented advances in computing systems with algorithmic developments to tackle emerging ‘big data’ problems in biomedical research brought on by the expansion of NGS technologies.

**Electronic supplementary material:**

The online version of this article (doi:10.1186/s12859-015-0736-4) contains supplementary material, which is available to authorized users.

## Background

Recent advances in next-generation DNA sequencing (NGS) technologies have increased the efficiency, reliability, and cost-effectiveness of sequencing, leading to ever-expanding amounts of high-quality data [1]. However, NGS reads have limited biological utility without reliable downstream processing and analysis, including read-quality assessment, alignment to a reference genome, assembly, variant identification, and individual genotyping [2]. While the tools for performing these steps have improved, processing a whole genome from generating and assessing the quality of the reads to calling and genotyping variants among a set of individuals remains an expensive and time-consuming component of sequencing studies. Furthermore, the reliability of genotyping from sequence data depends on accurate identification and accommodation of sequencing errors, which can be overcome to some degree by quality-control steps and by processing large numbers of sequencing reads simultaneously.

Here, we describe an efficient approach for obtaining high-quality variant calls and genotype assignments from a large set of whole human genomes sequenced on an Illumina HiSeq 2500 platform. Our approach exploits a ‘group calling’ framework to minimize genotype assignment errors that arise from an incomplete knowledge of sequencing error rates and inconsistent coverage of the genomes being processed. Essentially, group calling leverages reads obtained from more than a single individual’s genome in order to make more confident claims about the presence of a variant allele in any single genome.

This strategy can help mitigate false positive variant assignments but does have a few drawbacks, including the need to analyze individuals in a group with similar genetic backgrounds given varying allele frequencies and population-specific variants on a global scale [3]. In addition, the identification of *de novo* and very rare variants might be compromised with a group calling approach unless sensitivity to their possible existence is permitted. We showcase our strategy on 437 whole human genomes sequenced to ~35 × coverage and describe our implementation and results in detail.

Implementing variant calling workflows and computational schemes in an appropriate computing environment for studies involving large cohorts like ours is not trivial, as simply ingesting and storing the massive volume of data requires care and sophistication. In addition, designing and running a group calling workflow at large scale comes with unique challenges in high-throughput, data-intensive computing that are simply not present in small scale studies. In order to overcome the computational and storage requirements associated with the proposed approach and scale of our study, we leveraged computing facilities at the San Diego Supercomputer Center (SDSC) and in particular the Gordon computing system [4]. The Gordon system is unique among supercomputing systems and has many state-of-the-field features including a large amount of high-bandwidth memory per processing node, enough high-performance disk capacity to support all of the input, output, and intermediate data generated during a NGS analysis, and local flash-based storage to support the very intensive input and output operations necessary for implementing group calling workflows.

In order to process raw NGS reads and generate variant calls, we implemented the Broad Institute’s best practices pipeline recommendations, using the Genome Analysis Toolkit (GATK v2.7.2), including the *HaplotypeCaller* algorithm for calling variants [2, 5, 6]. This pipeline, like others, works in steps that include assessing the quality of the sequencing reads, aligning them to a reference genome, quality-control steps to improve variant identification, and assigning individual genotypes at each position in the sequence. Each of these steps has different computational requirements, including the number of concurrent computing nodes or ‘threads’ the step can use, how much memory it will need, how much immediate storage or ‘disk bandwidth’ it can consume, how many input/output (IO) operations per second (IOPS) it generates, and how much time it will take. In order to minimize cost, we optimized the steps involved to make them run as efficiently as possible on Gordon by running them in parallel across different components of the system where possible and appropriate. Furthermore, we were able to leverage several computing nodes containing large amounts of flash memory in addition to the default computing nodes on Gordon.

After benchmarking different strategies for variant calling we settled on using GATK’s *HaplotypeCaller* in groups of 20–24 genomes. This number of genomes per group was determined to balance computational efficiency with reliability of the variant calls. Furthermore, we found that variation in the ancestral backgrounds of the individuals used in a group influences the accuracy of variant calls and, consequently used groups consisting of individuals with similar ancestral backgrounds. Ultimately, our results provide insight into the computational challenges that researchers will face in the emerging era of ‘big data’ analytics in biomedical research and suggest that it is possible (and in fact critical) to incorporate sophisticated computing platforms with advanced algorithms and software to meet this challenge.

In this paper, we describe the resources used, variant calling approach, workflows, and the resulting variant calls from 437 whole genomes. First, we consider quality control steps pertaining to the reads and computing strategies to mitigate the computational burden associated with these steps. Next, we focus on the construction of the groups within which variant calling was pursued and, in particular, the influence of group size and the ancestry of the individuals in those groups. An assessment of the accuracy, sensitivity, and specificity of our group variant calling approach is provided by leveraging a gold standard human genome from the National Institute of Standards and Technology (NIST) [7]. We finally consider a comparison of our group variant calling with a simpler, oft-used single genome variant calling pipeline. We close with a discussion of additional computational aspects and limitations of our study as well as suggestions for future directions.

## Results

### Alignment and read processing

#### Mapping/Merging

**Fig. 1:**
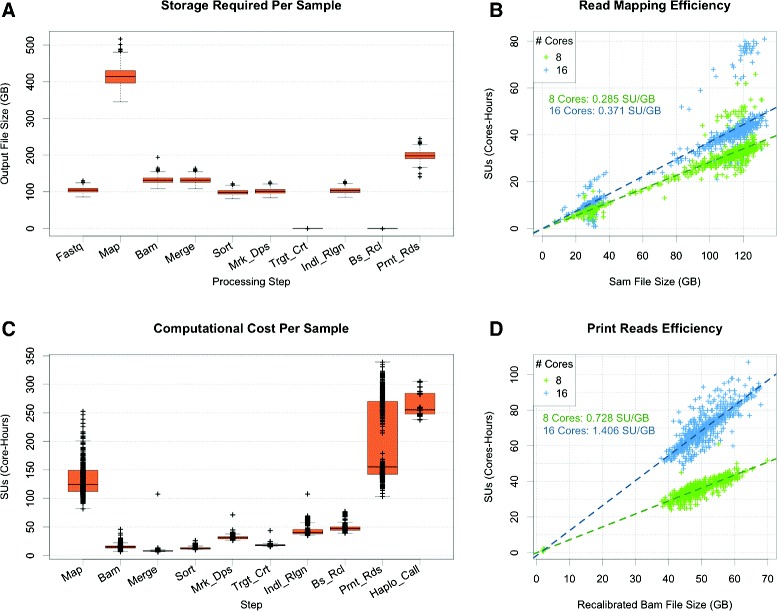
Technical summary of processing pipeline. **a** Storage requirement (GB) per sample for output file of each processing step. **b** Computational cost of mapping raw reads versus file size with 8 (green) or 16 (blue) computing threads. **c** Computational cost (SUs) per sample for each processing step. **d** Computational cost of *PrintReads* step versus file size with 8 (green) or 16 (blue) computing threads

When mapping reads with BWA, we attempted to use multi-threading over 16 cores per read group, but found that the mapping speed did not improve linearly with the number of computing threads (Fig. 1b). Therefore, we specified 8 threads per read group as a more cost-effective approach. The output of the mapping stage resulted in an uncompressed sequence alignment/map (SAM) formatted text file that is ~4 × larger than a compressed fastq file (~400–500 GB/sample; Fig. 1a, Additional file 1: Table S1). Subsequent compression of the SAM file resulted in a BAM file requiring only 25–30 % of the storage space, thereby reducing the overall footprint of aligned read files from ~175 TB to ~60 TB (Additional file 1: Figure S1A). The merged BAM file was comparable in size to the sum of the files containing individual read groups.

#### Sorting

When sorting a single whole human genome, upwards of 800 temporary files are generated, totaling up to 150 GB per genome. These files are repeatedly opened, read, written, and closed, imposing demanding IO performance. To fully utilize the sixteen CPU cores on each node, a single compute node must be capable of processing 2.4 TB of intermediate data spread across over 10,000 files. Unfortunately, the large parallel file systems available on most massively parallel supercomputers are designed to deliver high streaming bandwidth, not high IOPS [8, 9], and sorting several dozen genomes concurrently on Gordon’s Lustre file system resulted in severe performance degradation. To circumvent this issue, we utilized 2 “BigFlash” nodes, each with 4.4 TB of SSD flash storage.

The ability to write the temporary files to the flash space allowed us to maintain very efficient CPU utiliziation through the sort step by processing 32 genomes (16 per node) concurrently without being limited by IO performance, ultimately decreasing both the computational cost and wall time for this step. Without this ability, job packing would have been impossible and sorting the reads could have come at a needlessly high computational cost as a result of the IO limitations. The output file from the sorting step resulted in a slight compression from the unsorted input BAM file, requiring ~15 % less space than the unsorted file.

#### Realignment/Recalibration

When running, we allotted ~8 GB of memory per genome, allowing us to process 8 samples on a 64 GB node. Thus, 2 cores were designated for each instance of *MarkDuplicates*. This approach prevented the possibility of multiple job submissions exceeding the available memory and causing run errors. The *RealignerTargetCreator* step was similarly designated 8 GB of memory per genome and *IndelRealigner* steps utilized 12 GB of memory per genome, so 8 and 5 samples were run on a single node, respectively. This approach prevented memory allocation errors arising from memory exhaustion. *BaseRecalibrator*, on the other hand, could be run in parallel and was multithreaded to 8 cores per genome. Finally, *PrintReads* was run in parallel over 8 or 16 cores. When we established that specifying 16 computing threads yielded no performance increase, subsequent jobs were multithreaded over 8 cores (Fig. 1d).

The output from *RealignerTargetCreator* and *BaseRecalibrator* were negligible in size, while the output of *MarkDuplicates* and *IndelRealigner* were BAM files comparable in size to the sorted BAM file. The final, recalibrated BAM file was roughly double the size of the input BAM file and approximately 200 GB were required for each sample (Fig. 1a). Given the extraordinary amount of data being processed and generated, it is important to remove large, intermediate files after subsequent processing has been completed to minimize required storage resources. Without deleting intermediate files during the processing phase, each sample would require >1TB of storage space, totalling nearly half a petabyte for our cohort of 437 individuals (Additional file 1: Figure S1A). We suggest using correlations between input and output files sizes as one indication of successful completion of processing steps prior to deleting intermediate files from previous steps (Additional file 1: Figure S1B).

### Variant calling

#### Computing time

**Fig. 2:**
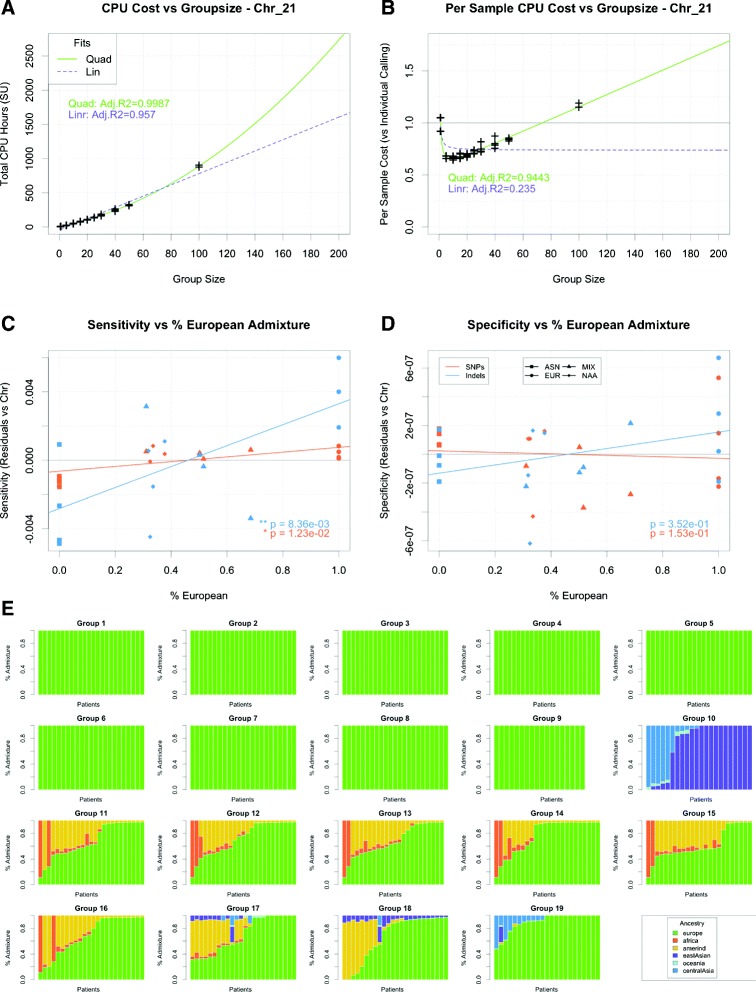
Variant calling assessment. **a** Total computational cost (SUs) of calling variants on chromosome 21 in varying group sizes. Adjusted R2 provided for linear (purple) and quadratic (green) fits. **b** Computational cost per sample (relative to individual variant calling approach) of calling variants on chromosome 21 in varying group sizes. Adjusted R2 provided, assuming linear (purple) and quadratic (green) fits for total computational cost. **c**, **d** Sensitivity **c** and specificity **d** of variant calls on NA12878 versus estimated proportion of European admixture within a group (normalized by chromosome). **e** Admixture estimates of groups used for variant calling

By comparing variant calling approaches using different group sizes, we show that the total computing time required to call variants for a group grows quadratically as the group size increases (Fig. 2a). Consequently, as group size increases, the "per sample" cost of calling variants sharply reduces at first, but then increases linearly when the group size is large (Fig. 2b). We estimate that the "per sample" cost of variant calling reaches a minimum for groups of ~15 individuals. Any computational cost benefit of group calling is lost when the group size reaches 75 and the cost increases linearly from there (~60 % increase per 100 individuals). For particularly large groups (>100), variant calls were made on only a fraction of chromosome 21 before the job timed out after 72 hrs (Additional file 1: Figure S3).

#### Accommodating ancestry

To further guide or grouping approach for variant calling, we called variants on portions of the NA12878 genome in groups with varying ancestral background (Additional file 1: Figure S4B). Concordance, defined as the fraction of variant calls made in all four ancestral test groups, amongst all four test groups tended to be higher after variant filtration was applied than in calls prior to filtering. Concordance was also higher for SNVs than indels (Additional file 1: Figure S5). For indels, over 87 % of variant positions were identified in at least 3 of 4 groups, while SNVs had greater than 90 % concordance amongst all test groups with over 95 % of SNV calls being made in at least 3 of 4 groups. To test the hypotheses that ancestral background of a group would affect the sensitivity and specificity of the variant calling process, we used a validated set of variant calls in high-confidence regions of the NA12878 genome [7]. We found evidence that the number of false negative SNV and indel calls was affected by the ancestral background of the group, but not the number of false-positive variants, suggesting that ancestral environment has a greater influence on sensitivity than specificity (Additional file 1: Figure S6). Because the NA12878 genome is of European ancestry, we investigated whether the estimated percent of European ancestry within a test group had an effect on the number of false-negatives or false-positives called on NA12878. The results were consistent with the initial results, indicating a positive correlation between sensitivity and the estimated fraction of European admixture within the test group for SNVs and indels after controlling for region of the genome (Fig. 2c). Again, there was no effect for the specificity of variant calls (Fig. 2d). Similarly, there was a positive correlation between overall accuracy and negative predictive value and fraction of European admixture, respectively, but no correlation with positive predictive value (Additional file 1: Figure S7). These results suggest that calling variants in groups of similar ancestry could result in fewer missed variant calls than a heterogenous group containing a high degree of admixture. Thus, in order to obtain high-quality variant calls in a computationally efficient manner, we called variants on the individuals in groups of 20–24 based on shared ancestry (Fig. 2e).

### Variant call comparison

#### Summary

**Fig. 3:**
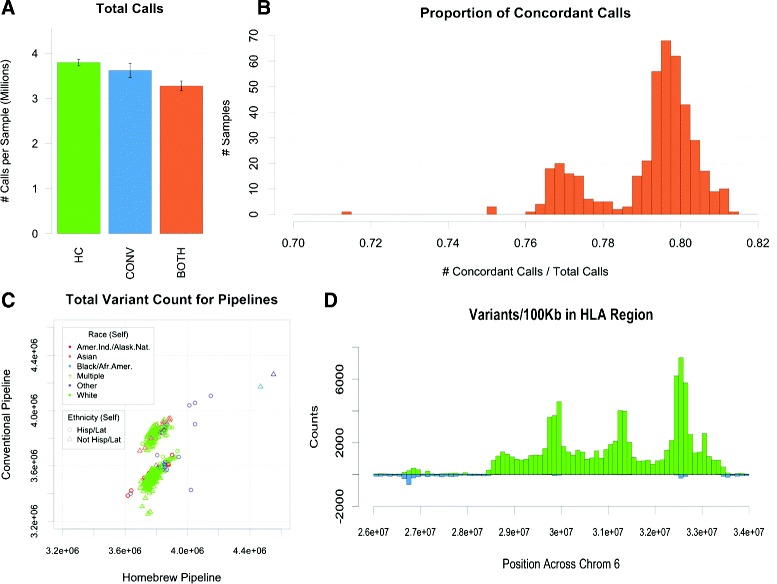
Comparison of conventional and HC variant calls. **a** Average number of variants called per genome by HC (green), conventional (blue), or both (orange). **b** Distribution of percent concordance between conventional and HC calls for 437 samples. **c** Number of variant calls made by conventional pipeline versus number of variant calls made by HC for each patient, colored by self-reported race. **d** Number of pipeline specific variant calls per 100 Kb in HLA region

The resulting variant calls were compared on an individual scale and as a cohort with variant calls made using the conventional pipeline. Our approach yielded 29,915,861 positions in which a SNV was identified across all 437 samples, while the conventional pipeline identified 30,790,918 SNV positions across 435 samples. Of those positions, both pipelines identified 27,247,530 (~81.4 %), while 3,543,283 (~10.5 %) were identified only by the conventional pipeline and 2,668,331 (~8.0 %) were identified only by our pipeline (Fig. 3a). Concordance between pipelines, defined as the intersection versus the union of variant sites for the pipelines, varied greatly for each individual as well, ranging from 71–82 %, and displayed a bimodal distribution (Fig. 3b). Surprisingly, our pipeline called, on average, more SNVs per genome despite identifying fewer unique positions, though the number of variants called on an individual varied between pipelines (Fig. 3a,c), and additional evidence of a batch effect is apparent in the conventional workflow.

The most glaring difference between variant call sets was the dearth of variant calls made by the conventional pipeline in the highly polymorphic human leukocyte antigen (HLA) region of chromosome 6 (Fig. 3d). The conventional workflow resulted in only a handful of calls in this region, while our own pipeline made tens of thousands of calls on the cohort. This may partially explain why our pipeline identified more SNVs per individual but fewer positions overall. This discrepancy could have staggering consequences in an association study, given the importance of the region in many autoimmune diseases. Other regions of inconsistency between call sets appeared most commonly near centromeres and telomeres. Often, both pipelines made variant calls in these regions that were inconsistent with one another (Additional file 1: Figure S9). This could be a result of poor local alignment in these particular regions. We further considered the prevalence of variant calls on each chromosome. After removing variants from the HLA region, the conventional pipeline resulted in more unique variant positions on each of the 22 autosomes and the X chromosome, while our own pipeline resulted in more unique calls on the Y chromosome and additional unmapped contigs, the latter of which were not included in the reference coordinates for the conventional pipeline (Additional file 1: Figure S10). This is consistent with the fact that the conventional pipeline yielded far more pipeline-specific variant positions overall.

**Fig. 4:**
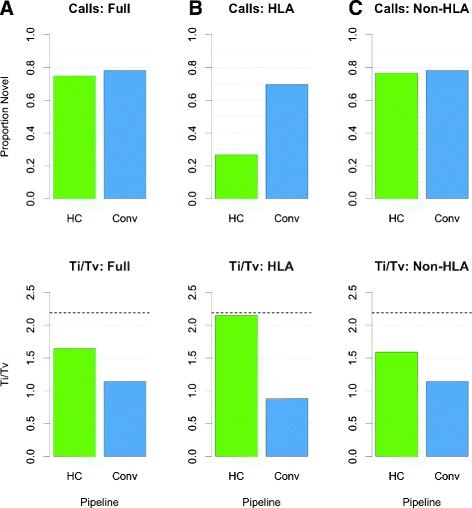
Summary of pipeline-specific variant calls. Proportion of novel variant sites and Ti/Tv ratios for pipeline-specific calls. **a** Variants across the genome. **b** Variants in HLA region. **c** Non-HLA variants

In addition to differences in the location of variant calls between the two call sets, we considered differences in the frequency, novelty, and classification of variant calls made exclusively by each pipeline. Of the variant calls made by a single pipeline, a slightly higher proportion of our calls were rare (minor allele frequency (MAF) <1 % in our cohort), while a slightly higher proportion of calls made by the conventional pipeline were low-frequency (MAF 1–5 %). A similar proportion of calls made exclusively by each pipeline were common (MAF >5 % (Additional file 1: Figure S11). Furthermore, a larger fraction of the calls made specifically by the conventional pipeline were novel (not identified in dbSNP) than those made only by our own pipeline. The same pattern holds true when considering only variant calls outside of the HLA region. In the HLA region, the majority of variant calls made exclusively by our pipeline were known variants, while the majority of the relatively few calls made in that region only by the conventional pipeline were novel (Fig. 4b). Finally, calls made exclusively by our pipeline had a transition to transversion (Ti/Tv) ratio much closer to the 2.19 expected for a whole human genome than did calls made by the conventional pipeline. The same trend is true for variants in the non-HLA region of the genome, and the difference is especially apparent for variants within the HLA region (Fig. 4c). These data suggest that the variant calls made by our pipeline are generally more consistent with what is expected than those made by the conventional pipeline.

#### Impact

**Fig. 5:**
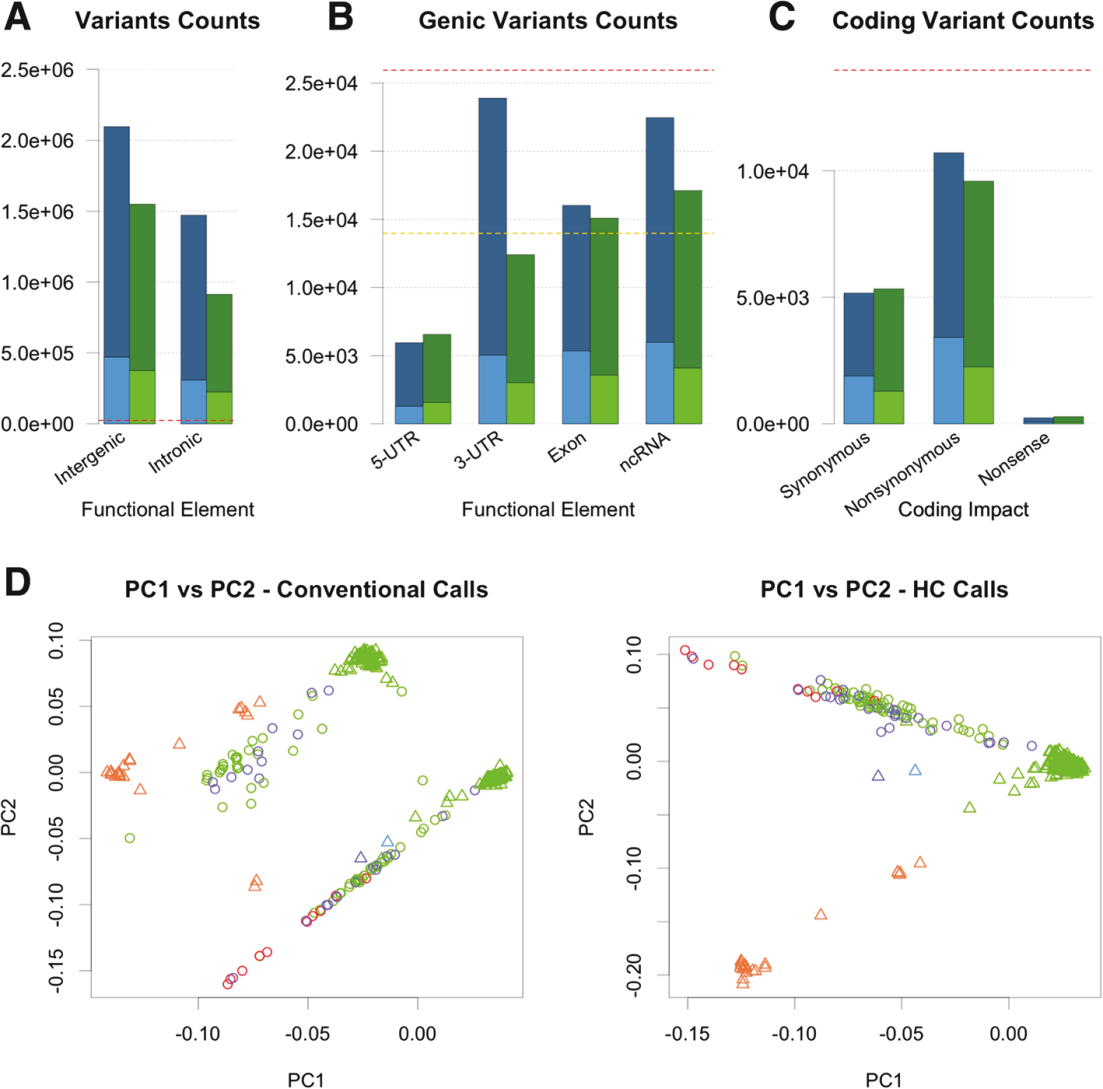
Impact of variability between pipelines. **a** Number of intergenic and intronic pipeline-specific variants. **b** Pipeline-specific variants in exonic, UTR, and non-coding RNA elements. **c** Functional impact of pipeline-specific protein-coding variants (blue = Conventional; green = HC; light = Novel; dark = Known). **d** Principal components 1 and 2 calculated from genotypes (MAF >1 %) using variants calls from conventional (left) or HC (right) pipelines. Individuals coded by self-reported Race (red = American Indian/Alaska Native; orange = Asian; yellow = Multiple; green = White; blue = Black/African American; purple = Other) and Ethnicity (circle = Hispanic/Latino; triangle = Not Hispanic/Latino)

We further assessed the location of variant calls relative to functional elements in the genome and the predicted functional consequences of those variants. Excluding variants in the HLA region, we discovered that there were more calls specific to the conventional pipeline in intergenic, intronic, exonic, 3’ untranslated region (UTR), and noncoding RNA (ncRNA) regions than calls made exclusively by our pipeline. This effect was most striking for 3’ UTR regions, while our pipeline actually resulted in slightly more calls in 5’ UTR regions (Fig. 5a,b). When we normalize to the total number of variants uniquely called by each pipeline, we see that our group calling strategy resulted in a higher proportion of calls landing in intergenic, exonic, 5’ UTR and ncRNA regions than the conventional pipeline-specific calls (Additional file 1: Figure S12A). In assessing the coding impact of the pipeline-specific variants in non-HLA regions, we found that the conventional pipeline made more calls predicted to result in nonsynonymous amino acid changes, while calls specific to our pipeline resulted in more synonymous and nonsense variant calls (Fig. 5c). Spurious variant calls would not be subject to real-world natural selection, so one might expect to find an enrichment of deleterious variants in a set of false-positive variant calls; however, this would need to be validated with functional assays. It should be noted that our group calling workflow-specific calls contained a higher proportion of, albeit fewer, variant calls in all three categories (Additional file 1: Figure S12B).

To further evaluate the accuracy of our variant calls relative to those made by the conventional pipeline, we performed principal component analysis (PCA) and compared the clusters formed by plotting the first two PCs against one another. These measures have been shown to represent the geographical separation amongst different populations, with individuals from similar ancestral backgrounds clustering together. While variant calls made by our pipeline result in clusters that recapitulate self-reported race and ethnicity, calls made with the conventional pipeline show a batch effect with no biological interpretation. Two distinct European clusters are present along with two distinct clusters of Asian and Hispanic individuals extending away from their respective European clusters (Fig. 5d). These results suggest that some non-biological process is resulting in a large proportion of genomic variation in this cohort when the variants from the conventional pipeline are considered. Using variant calls that do not reflect the biological reality of the patient would lead to spurious results in an association study.

Our results speak to the importance of the sophistication of variant calling workflows in obtaining high-quality data for use in association studies. Furthermore, we highlight the importance of efficiency in large-scale sequencing studies and describe an approach that yields reliable variant calls in a computationally efficient manner.

## Discussion

While traditional chip-based, genome-wide association studies (GWAS) have proven to be useful in biomedical research settings, they have limited utility for discovery of rare variant associations. Due to the high cost of NGS technologies relative to targeted genotyping arrays, whole-genome sequencing (WGS) has seen limited use in GWAS. However, since the first human genome was sequenced and assembled just over a decade ago, the cost of sequencing has fallen dramatically [1, 10, 11]. As a result, the amount of NGS data being produced is increasing rapidly with hundreds of thousands of whole human genomes likely to be sequenced over the next few years. The ability to efficiently and reliably obtain an individual’s genotype at any location in the genome via sequencing has motivated genome-wide association studies that leverage DNA sequence data rather than traditional chip-based, targeted genotyping information [12]. The need for larger studies that consider rare and *de novo* variants obtained via NGS is especially apparent if one wishes to investigate the contribution of such variants to complex disease. This will result in extraordinary amounts of raw data in need of storage, processing, and subsequent analysis and interpretation.

NGS technology, with its inherent limitations in library preparation and machine errors, results in errors and biases in the sequence data generated. Furthermore, it relies on relatively short reads that must be aligned to a reference genome and processed before variants are called and genotypes are assigned to individuals. Consequently, the variant identification and genotyping results can vary depending on the quality and complexity of the variant calling workflow and algorithms used. Variant calling and genotyping steps require computational resources beyond the capabilities of a typical desktop system. For example, more sophisticated variant calling workflows tend to include additional recalibration and local realignment steps that better account for sequencing errors and natural genetic variant and result in more reliable genotype assignment, but require more computing resources than simpler pipelines. Nevertheless, the quality of data is of the utmost importance since unreliable genotyping can lead to spurious disease associations. As associations are identified, annotations made, and databases built, the need for high-quality data to provide meaningful insight into the genetic variants that are accurately linked to phenotype cannot be stressed enough.

In this light, it is critical to improve the efficiency with which NGS data are processed. Much of that processing is likely to be accomplished using cloud-based services such as those offered by Amazon, Microsoft, or Google. Computational core hours are the effective currency of most supercomputing platforms, and often have a direct translation to actual costs (e.g., core hours run between $0.05 and $0.11 per core-hour on commercial clouds, though supercomputing platforms tend to be more cost-effective). In addition, while computational inefficiencies can be tolerated with smaller studies, as studies recruit more patients to undergo WGS, such inefficiencies can accumulate and cost tens of thousands of dollars in computing time. As such, minimizing the number of core hours used is critical to ensuring overall cost-effectiveness of any large-scale study, especially population-based studies using WGS. Our experiences and results leveraging a group calling approach to variant calling and genotyping implemented on a supercomputer could provide further motivation to evaluate costs and efficiencies in handling NGS data at scale. By leveraging the Gordon computing system at SDSC, we assembled a processing and variant calling pipeline that makes efficient use of Gordon’s unique capabilities.

Several steps allow multi-threading of processes, but performance often does not scale linearly with the number of cores used. As a result, utilizing fewer cores may prove to be more cost-effective in some instances, as demonstrated by our experimentation with BWA *mem* and GATK’s *PrintReads*. Less parallelized approaches may prove more computationally efficient, but increase walltime. Thus, one should find a balance between computational efficiency and speed depending on one’s immediate goals. For example, identifying variants on a tumor in order to guide treatment should focus on a quick turnover of the data while a genome-wide study of large cohorts should aim for computational efficiency.

Additionally, when single jobs could not be multi-threaded, we packed several similar jobs onto a single node in order to avoid being charged for idle cores. We found that grouping together many logical tasks of the same pipeline stage (e.g., marking duplicate reads) as a single job submission resulted in significantly greater computational efficiency than submitting all pipeline stages as a single job. Given the fact that many large clusters (with 10,000 cores or more) only accept jobs that request resources in whole-node increments (e.g., 16 cores and 64 GB RAM on the SDSC’s Gordon), grouping logical tasks of the same pipeline stage offered two-fold benefit over submitting a complete multi-stage pipeline as a job. First, all tasks of a common pipeline stage exhibit generally uniform resource requirements in terms of wall time and core and memory utilization, which ensures that one task does not take significantly longer than all others within a single job and leave the allocated node largely idle. Furthermore, specifying more cores than are necessary for a given step can lead to a tremendous increase in computing costs. If this issue is not addressed, one could wastefully consume hundreds of thousands of hours of computing time when scaling up to large cohorts of whole human genomes (Fig. 1c, Additional file 1: Figure S2). For jobs that required more memory, the limiting factor was the 64 GB of memory per node. Thus, if a job required 8 GB of memory to process a single file, we specified 2 cores for each instance, limiting the number of instances that could be run in that job while making our pipeline very reliable and minimizing the number of failed jobs resulting from memory allocation errors. Second, launching groups of common tasks together allows these stages to reserve specialized resources such as BigFlash nodes or large memory nodes only for the stages where they are essential. While they promote computational efficiency, the use of these specialized resources may prove to be a bottleneck in processing large cohorts if they are not readily available. This job packing approach that stresses similar computational requirements of each step saves tremendously on computing costs. Processing a single genome from start to finish on a single full node would result in idle cores and result in a 16-fold over charging during certain steps in the pipeline. Thus, we again emphasize that efficient and balanced use of software and hardware resources on advanced computing platforms is paramount.

When calling variants on 437 whole human genomes in groups, we also considered group size and ancestral background. It has been previously suggested that all samples should be considered in one large group during variant calling; however, the computational cost of calling a large cohort simultaneously makes this approach prohibitively expensive. We found a quadratic increase in overall computing time for variant calling as group size increased and a linear increase in "per-sample" computing time when the group is large. As a result, we predict that calling variants on our entire cohort in one group would result in a computational cost ~5 × greater than our approach of calling variants in groups of 20–25 individuals (Fig. 2b). Similarly, calling variants on individuals would have been roughly ~40 % more expensive than our approach and would not have taken advantage of the increased power of group calling to detect variation in regions of low coverage.

To avoid the prohibitively long compute time, while retaining the power associated with group-calling, we explored approaches to grouping samples from a large, admixed population. Our results suggest that calling variants in a group with genomes from a similar ancestral background increased the sensitivity, but had no discernible effect on the specificity of variant calls. With this in mind, we placed samples into groups of similar ancestral background and called variants in 19 separate groups. When more efficient algorithms for variant calling are developed and it is possible to identify variants on a large cohort at a reasonable computational cost, one may consider the advantages and disadvantages of using a single large group, but at the moment that approach remains computationally prohibitive.

The variant calls from our group-based pipeline differed significantly from those provided to us from a conventional pipeline. As opposed to the the conventional workflow used to initially call variants, we included recalibration and realignment steps designed to increase the accuracy of subsequent SNV and indel calls. Furthermore, we employed the *HaplotypeCaller* command, which utilizes a *de novo* assembly approach in assigning genotypes to an individual’s genome. This feature is complemented by a group-calling approach that considers haplotypes from several ancestrally similar samples at once, thereby increasing the power to detect variation in regions of low coverage for a single genome. The differences between the two pipelines could yield significantly different results in association studies.

## Conclusions

When comparing the calls made by our group calling workflow and the previous workflow, we found that the previous set of calls generated a dearth of variant calls in the highly polymorphic HLA region of chromosome 6, while our own pipeline identified tens of thousands of variants in that region. In assessing the reliability of calls in this region and throughout the genome, we utilized the Ti/Tv ratio as a measure of reliability. The variant calls made by our pipeline had a Ti/Tv ratio closer to the expected value of 2.19, supporting our claim of greater reliability from our own pipeline. Furthermore, when assessing the principal components and the distributions of the genotype assignments from the conventional pipeline, we identified a large batch effect that is likely an artifact from the workflow. When utilizing the same raw data processed through our own pipeline, the batch effect disappeared, and the principal component analysis accurately clustered samples by their self-reported biogeographical ancestry.

As a result of our experiences, we have several recommendations for processing large numbers of genomes (Table 1). First, consider parallelizing in a manner that reduces computational cost rather than overall speed, as demonstrated by mapping on 8 rather than 16 cores (Fig. 1b). This may result in moderately slower turnover of data, but can save money by reducing computing time. Second, pack jobs by logical tasks rather than by individual in order to avoid by charged for idle cores during steps that cannot be multi-threaded. Third, consider memory allocation when submitting jobs in order to reduce the number of errors and make your pipeline more reliable. The number of commands submitted simultaneously to a single node will, consequently, depend on the size of the node and the memory allocated for each job. Finally, when calling variants, group individuals in a way that promotes efficiency and accuracy. We show that calling variants on individuals in groups of 20–25 individuals of similar ancestral background is both computationally efficient and produces reliable variant calls. Overall, our results suggest that our group calling approach implemented on a supercomputer yields high-quality variant calls and genotype assignments in an efficient manner and highlights the need for sophisticated computational strategies in the analysis of the large numbers of human genomes that will be sequenced in the coming years.

**Table 1:** Recommended job packing approach for best practices pipeline. Assumes node with 16 processors and 64 GB of memory

Step	Tool	Memory per	Cores per	Commands
		command (GB)	command	per node
Map	BWA	32	8	2
Bam	Samtools	4	1	16
Merge	Samtools	4	1	16
Sort	Samtools	4	1	16
MarkDuplicates	PicardTools	7	2	8
TargetCreator	GATK	7	2	8
IndelRealigner^*^	GATK	12	3	5
BaseRecalibrator	GATK	30	8	2
PrintReads^*^	GATK	30	8	2
HaplotypeCaller	GATK	60	16	1

^*^Smaller memory allocation and more samples per node may prove more computationally efficient

## Methods

### Samples

Our sequencing study included 437 of the RA subjects enrolled in GO-FURTHER clinical trial [13, 14]. Study design and protocol were approved by local Institutional Review Boards at each site. Trial patients who did not give approval for use of tissue in sequencing study were omitted from the sequencing study and all subjects included in sequencing study provided informed consent upon entering the clinical trial. No additional approval for sequencing study was required. Whole blood samples were collected according to approved trial protocol. Details can be found at http://www.clinicaltrials.gov (ID: NCT00973479) and previous publications [13, 14].

### Whole-genome sequencing

Whole blood samples were processed at the Beijing Genomic Institute (BGI) for DNA sequencing. Raw 90-base pair (bp), paired-end read sequences were produced using an Illumina HiSeq 2500 platform. The average depth of haploid coverage by mapped reads was ~35 ×. The average genome coverage is 99.4 %. Reads were initially mapped using BWA *aln* algorithm and variants were called on individuals using SOAPsnp with a conventional pipeline that did not include recalibration or realignment steps. Subsequent variant calling utilized the same reads produced by BGI.

### The Gordon supercomputing system

Gordon was designed to address data-intensive problems, such as those that are prevalent in biomedical research [9]. First, Gordon contains 1024 compute nodes, each with 16 Intel Sandy Bridge cores and 64 gigabyte (GB) of double data rate (DDR) dynamic random-access memory (DRAM) capable of an aggregate 64 GB/s memory bandwidth. Second, two high-performance Lustre file systems are tightly integrated with Gordon; a 1.6-petabye (PB) ‘scratch’ file system provides up to 100 GB/s aggregate throughput from the compute nodes, while a second, 1.4 PB ‘projects’ file system is available for longer term storage. Furthermore, 300 GB enterprise solid-state disks available on each compute node are capable of delivering 220 MB/s of bandwidth and 37,000 IOPS each, aggregated into 4.4 terabyte (TB) software redundant array of independent disk (RAID) arrays capable of 1.6 GB/s throughput and 319,000 IOPs. Finally, Gordon includes a second quad data rate (QDR) InfiniBand fabric exclusively used for storage communication [4]. For challenging data intensive problems such as ours, we were able to leverage several “BigFlash” computing nodes, each consisting of 4TB of SSD flash memory for ultra-fast reading and writing of temporary data.

### The group calling workflow

The input data set consisted of compressed fastq and vcf files for the 437 individuals in our study with on average 100 GB per sample. As a result, the initial data storage requirements for the approximately 2,100 pairs of files containing compressed reads were almost 47 TB. Below we outline computational details of the various steps in our workflow.

#### Alignment and read processing

The reads for each read group were mapped to the reference human genome hg19 individually using the Burrows Wheeler Aligner (bwa *mem*) [15, 16]. Each read group was mapped using 8 or 16 cores concurrently, resulting in parallelization over 32–64 computing threads per genome during the mapping phase. After the reads were aligned to the reference genome, additional processing steps were implemented to improve the reliability of subsequent variant calls. First, mapped SAM files for each read group were compressed using the Samtools *sort* command, producing binary alignment/map (BAM) files [17]. The Samtools *merge* command was subsequently used to merge read groups from a single sample into one BAM file per genome. Since the Samtools *view* and *merge* commands cannot be multi-threaded and require relatively little memory to execute, each file could be processed using a single core. Thus, 16 commands were executed simultaneously on a single node.

Next, the bam file for each sample was sorted by genomic coordinate. The Samtools *sort* command similarly requires relatively little memory and can be run with a single core per sample; however, the writing of many temporary files limits performance on a standard node. We utilized two “BigFlash” nodes, each containing 4.4 TB of local solid-state device (SSD) flash storage to which the ~800 temporary files per genome could be written. We executed the *sort* command on 16 samples per “BigFlash” node, writing all files to the SSD flash memory and then copying the final, sorted BAM file into the long-term storage space.

After the BAM files were sorted by genomic coordinate, the Picard Tools *MarkDuplicates* command was used to flag duplicate reads that resulted from PCR amplification of the DNA which were subsequently discarded (http://broadinstitute.github.io/picard). Because of the relatively larger memory requirement, only 8 samples were processed on a 64GB node, effectively allocating two cores and 8GB of memory to each sample. Following execution of *MarkDuplicates*, local realignment steps were implemented using GATK’s *RealignerTargetCreator* and *IndelRealigner* tools in order to improve mapping quality, reduce false-positive SNP calls, and increase true-positive indel calls. The *RealignerTargetCreator* step, similar to *MarkDuplicates*, required more memory than earlier processing steps and was run with 8 samples per node, while the *IndelRealigner* step was specified with a slightly greater memory allocation still and was, consequently, run with only 5 samples per 64 GB node.

Finally, the realigned BAM files were run through a base recalibration step to account for systematic variation and bias in the sequencing process. GATK’s *BaseRecalibrator* tool modifies base quality scores for each read by considering the original quality score, read group, machine cycle, base-position within the read, and flanking bases in the read. This step can be parallelized, using multiple computing threads for each sample; however, the recalibration step should be implemented on the entire genome rather than a portion of it (i.e., separate recalibration calculations for each chromosome). Therefore, each whole genome was multi-threaded to 8 cores. Using *PrintReads*, each sample was output to 4 separate BAM files (chr1–3, 4–8, 9–14, 15-End) to facilitate parallelized variant calling and to decrease the wall time for outputting the final BAM files. Thus, each sample was output using 4 separate instances of *PrintReads*, each specifying 8 or 16 cores.

#### Group variant calling and variant filtering

To perform variant calling on the 437 genomes, we used GATK’s *HaplotypeCaller* algorithm leveraging a group calling approach in which aligned reads from multiple samples are considered together and genotypes are generated for the individual genomes in the group simultaneously. To determine the grouping strategy that achieved optimal computational efficiency and accuracy, we called variants in many test groups of varying size and ancestral background. To assess the computational burden of calling variants in groups of different sizes, we randomly selected patients from our cohort of 437 individuals, placed them in groups of varying sizes (1, 5, 10, 15, 20, 25, 30, 40, 50, 100, 200, 300, 400, 437), called variants on chromosome 21 using GATK’s *HaplotypeCaller*, and tracked their progress over time. Variant calling on these test groups was parallelized to 16 computing threads for each group.

To assess the influence of admixture within a group on variant calls, we estimated the degree of admixture of each sample from 6 biogeographical groups using variant calls from the conventional pipeline at ancestrally informative positions [18] (Additional file 1: Figure S4A). The required ancestry-informative variants could be similarly identified at a relatively small computational cost by implementing GATK’s *UnifiedGenotyper* algorithm at pre-specified locations on all samples prior to calling variants on the entire genome. To determine the best way to group samples for our cohort of 437 individuals, we called variants on chromosomes 14, 17, 20, and 22 of the European NA12878 genome in test groups with varying ancestral backgrounds and compared calls to established high-confidence variant and reference calls [7]. Admixture estimates were used to place samples into test groups of varying ancestry, including entirely European, entirely Asian and Oceanic, admixed African and Native American, and highly admixed (Additional file 1: Figure S4B). We determined the concordance and accuracy of variant calls made on NA12878 under these differing ancestral conditions for SNPs and indels. Because variants were called on a single chromosome for each test group, we implemented a variant filtration method utilizing hard thresholds on various quality metrics rather than using a more sophisticated variant recalibration approach. We then calculated the sensitivity, specificity, accuracy, PPV, and NPV of the variant calling process in each of the ancestral test groups and regressed against the estimated fraction of European admixture in each group, controlling for chromosome on which the variant calls were made.

To perform group variant calling, we placed the 437 genomes into groups of individuals with similar biogeographical ancestry (Fig. 2c). Each group contained 20–24 individuals, which provided the increased power for variant detection, while avoiding the prohibitively long compute time associated with large group variant calling. SNVs and indels were called for each group using the multi-sample feature of GATK’s *HaplotypeCaller*. Variants for each group were called in four parallel computing jobs, containing chromosomes 1–3, 4–8, 9–14, and the remaining chromosomes and contigs. This approach, along with multi-threading each job onto 16 computing cores, decreased the wall time necessary to complete the variant calling step.

Once raw variant calls for each group were obtained, the files within a group were concatenated. Variants were then subjected to GATK’s *VariantRecalibrator* and *ApplyRecalibration* steps for SNVs and indels, separately. This approach, rather than drawing hard thresholds on quality metrics, builds a sophisticated model that considers depth of coverage at a variant site, the average quality per read at a variant site, evidence of strand bias, mapping quality, and position within the read. As expected, groups containing greater genetic diversity identified more variant positions relative to the reference genome (Additional file 1: Figure 8). Finally, variants from each group were merged into a single vcf file and any variant position designated as "missing" was regenotyped as a homozygous reference genotype.

## Additional file

Additional file 1
**Supplemental material.** PDF file containing supplemental figures and descriptions (PDF 5120 kb)

## Abbreviations

NGS: Next generation sequencing
SDSC: San Diego Supercomputer Center
GATK: Genome Analysis Toolkit
IO: Input/output
IOPS: IO operations per second
NIST: National Institute of Standards and Technology
BGI: Beijing Genomic Institute
DDR: Double data rate
GB: Gigabyte
PB: Petabyte
DRAM: Dynamic random-access memory
TB: Terabyte
RAID: Redundant array of independent disk
QDR: Quad data rate
BWA: Burrows Wheeler Aligner
SAM: Sequence alignment/map
BAM: Binary alignment/map
SSD: Solid-state device
SNV: Single nucleotide variant
Indel: Insertion/deletion
HLA: Human leukocyte antigen
MAF: Minor allele frequency
Ti/Tv: Transition/transversion
PCA: Principal component analysis
UTR: Untranslated region
ncRNA: Non-coding RNA
GWAS: Genome-wide Association Study
WGS: Whole-genome sequencing
