# Supplementary material for: Group-based variant calling leveraging next-generation supercomputing for large-scale whole-genome sequencing studies
Source: BMC Bioinformatics. 2015 Sep 22;16(1):304. doi: 10.1186/s12859-015-0736-4 (PMC4580299; doi:10.1186/s12859-015-0736-4)
Supplement: Additional file 1 — Supplemental material. PDF file containing supplemental figures and descriptions (PDF 5120 kb) [file 12859_2015_736_MOESM1_ESM.pdf]

Supplementary Material

Standish KA et al.

Group-Based Variant Calling Leveraging Next-Generation Supercomputing for Large-Scale Whole-Genome Sequencing Studies

Contents:

Main Figures 1-5 (no captions)
Table 1 (no caption)
Supplementary Figures 1-10 (w/ captions)
Supplementary Table 1 (w/ caption)

MAIN FIGURE 1:

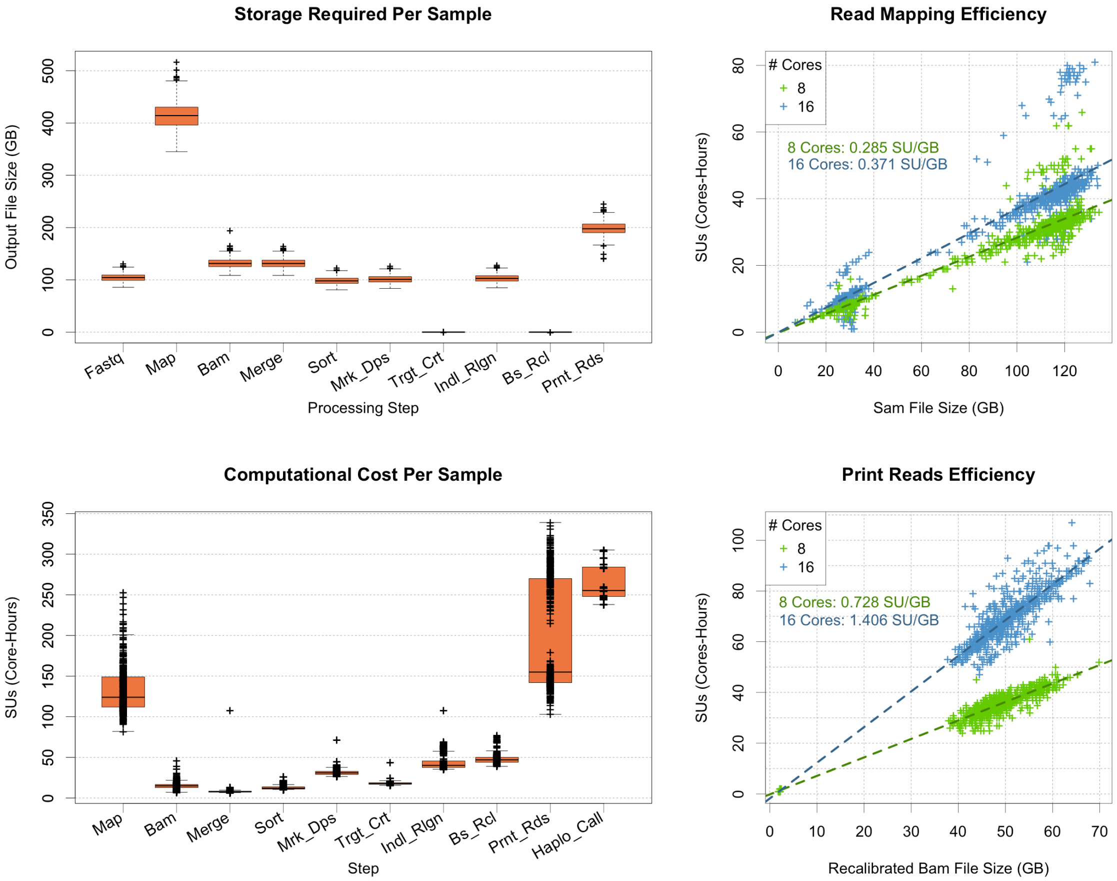

MAIN FIGURE 2:

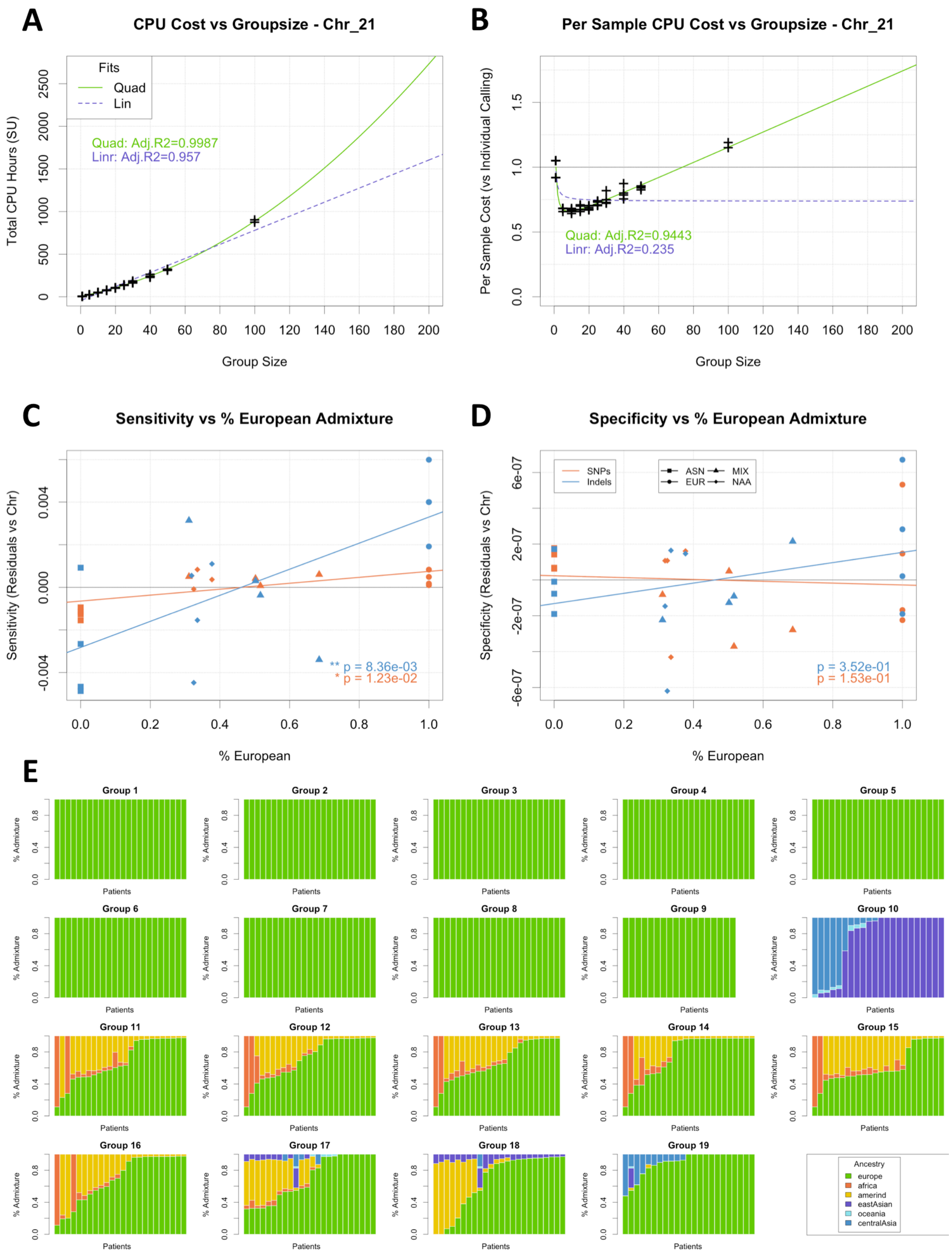

MAIN FIGURE 3

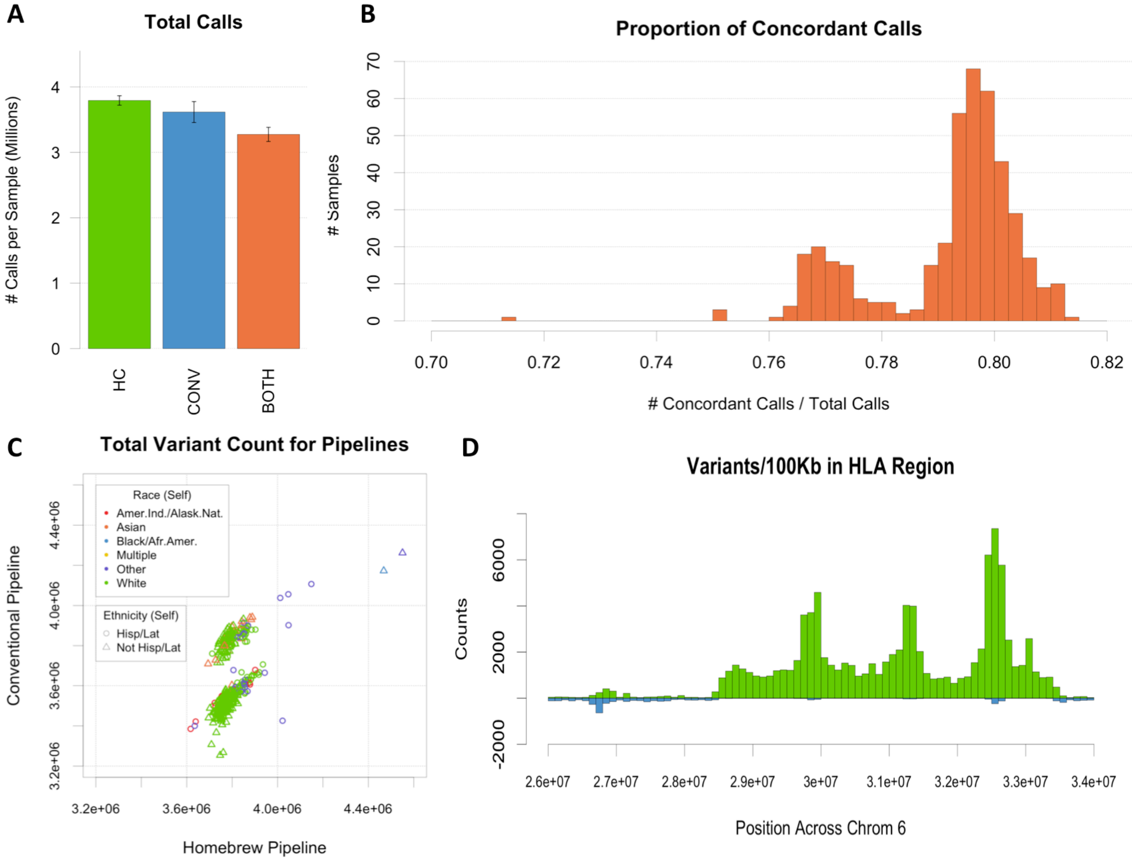

MAIN FIGURE 4

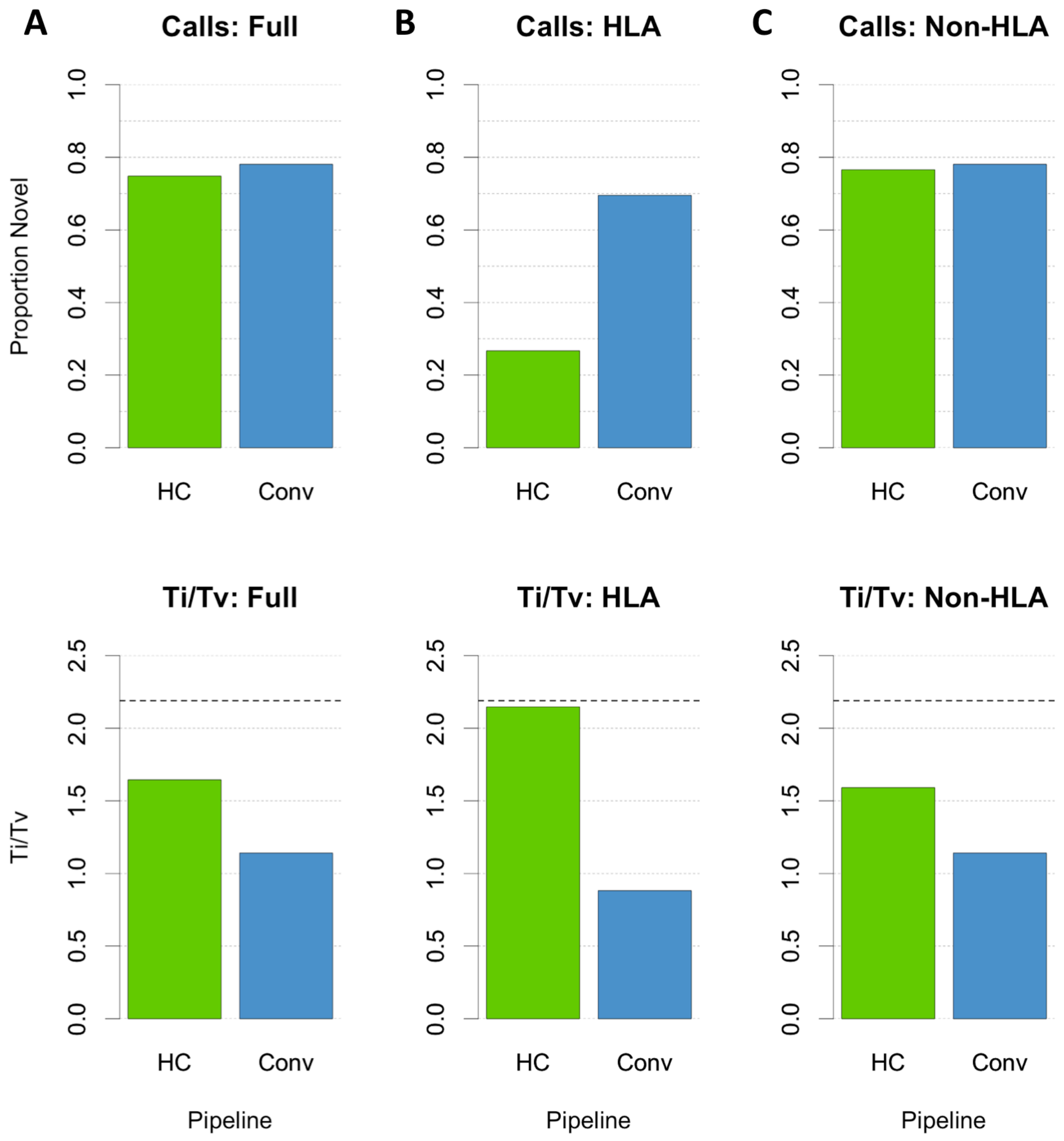

MAIN FIGURE 5

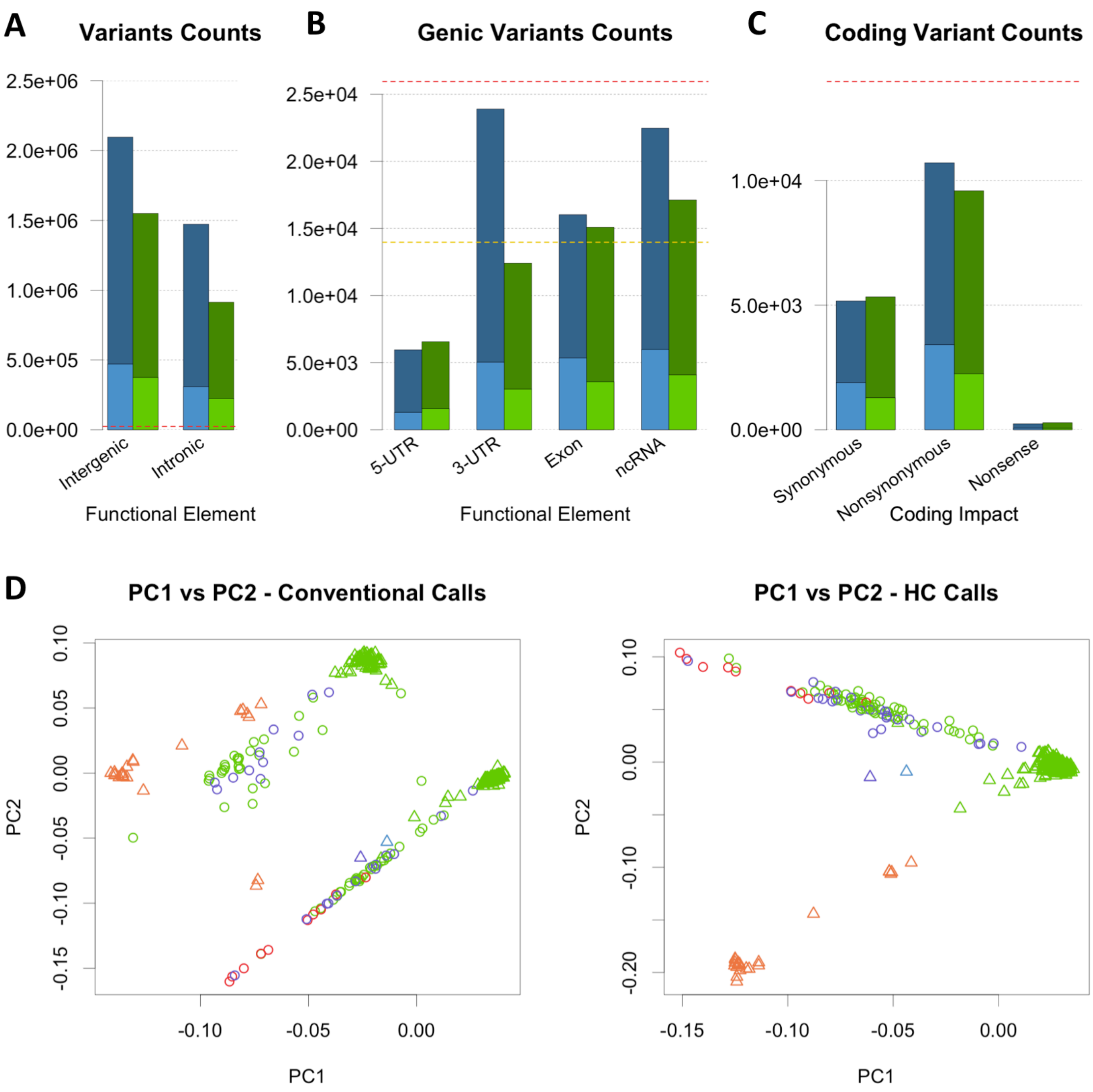

TABLE 1

Step	Tool	Memory per Command (GB)	Cores per Command	Commands per Node
Map	BWA	32	8	2
Bam	Samtools	4	1	16
Merge	Samtools	4	1	16
Sort	Samtools	4	1	16
MarkDuplicates	Picard	8	2	8
TargetCreator	GATK	8	2	8
IndelRealigner	GATK	12	3	5
BaseRecalibrator	GATK	32	8	2
PrintReads	GATK	32	8	2
HaplotypeCaller	GATK	64	16	1

SUPP FIGURE 1

A

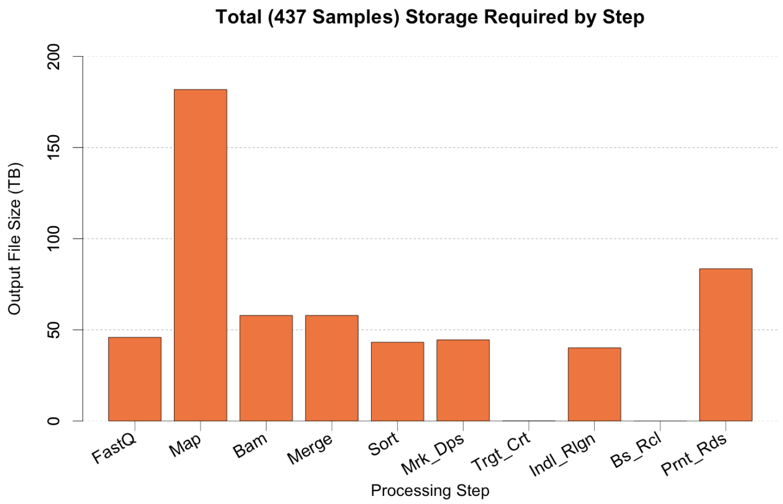

B

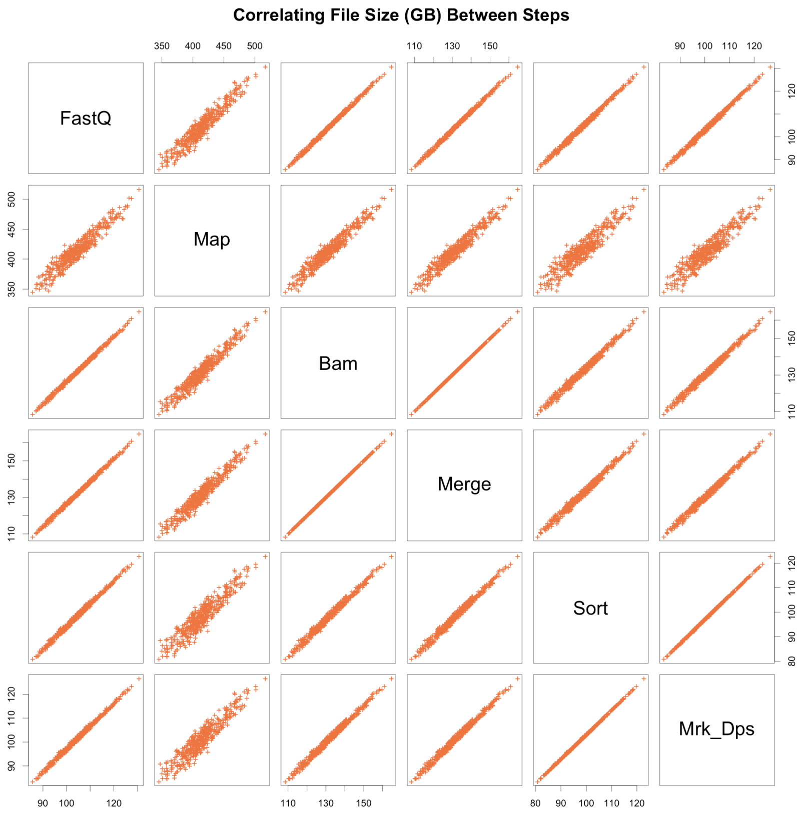

Supp. Figure 1:
(A) Total storage requirement for all samples in cohort, by processing step.
(B) Correlation between input and output file size for each processing step

SUPP FIGURE 2

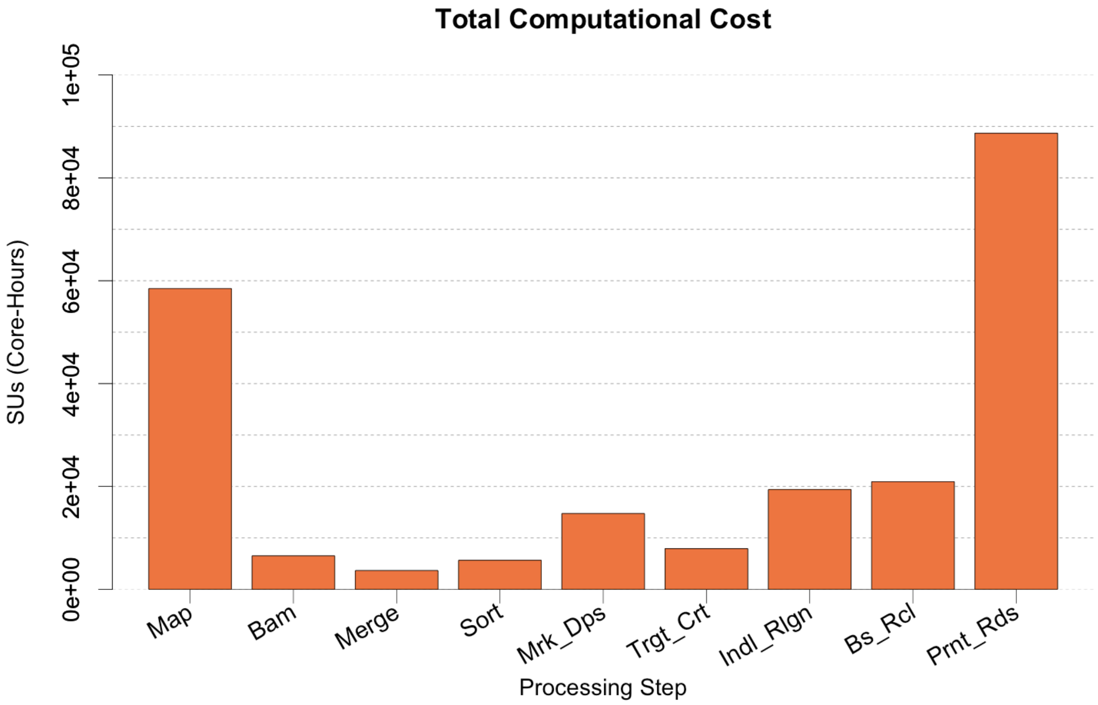

Supp. Figure 2:
Total core-hours (SUs) used for preparing reads for variant calling, by processing step.

SUPP FIGURE 3

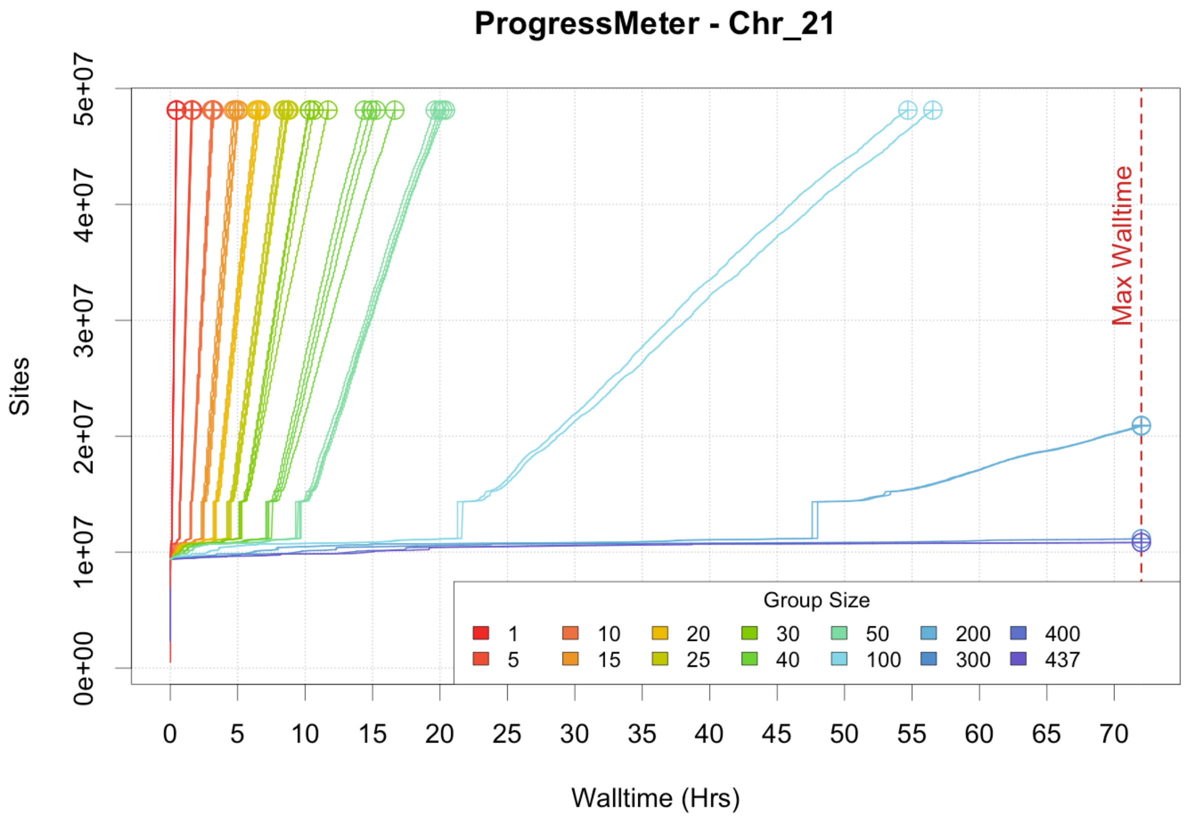

Supp. Figure 3:
Wall time (in hours) to call variants on chromosome 21 for a groups of varying sizes.

SUPP FIGURE 4 (A&B)

A

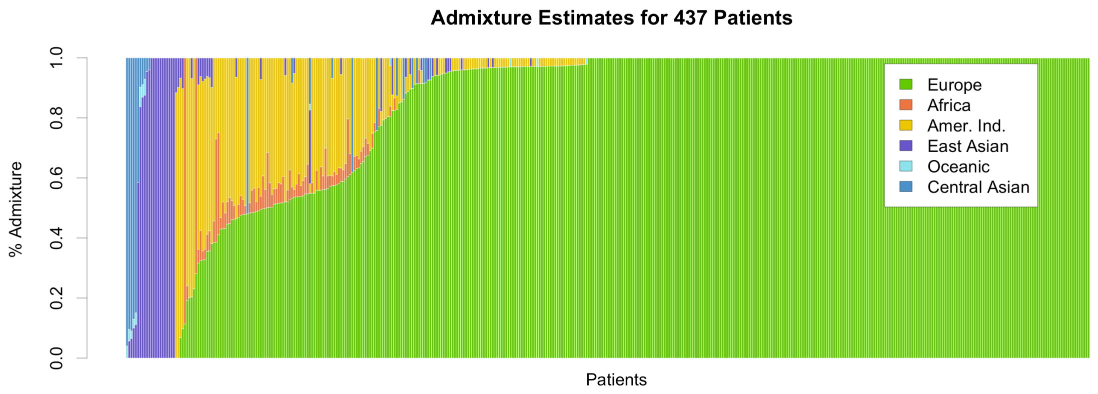

B

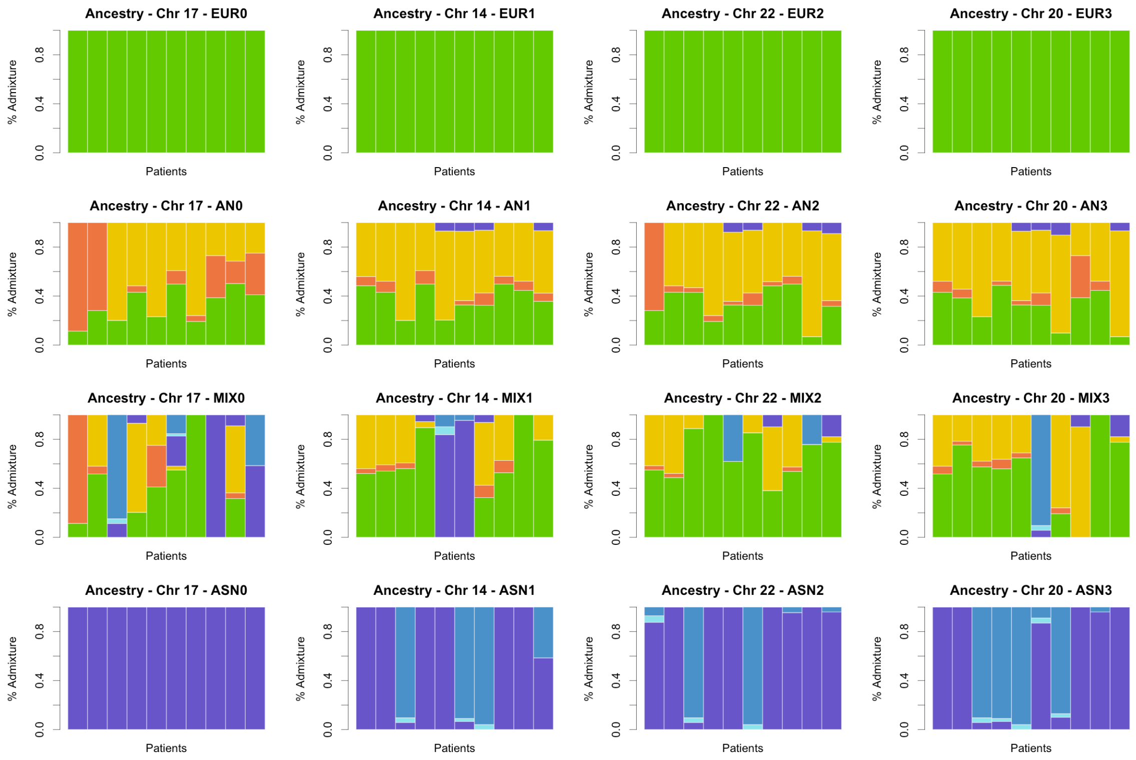

Supp. Figure 4:

(A) Admixture estimates obtained for 437 patients in cohort

(B) Admixture estimates for each test group in which variant for NA12878 were called on specified chromosome

SUPP FIGURE 5

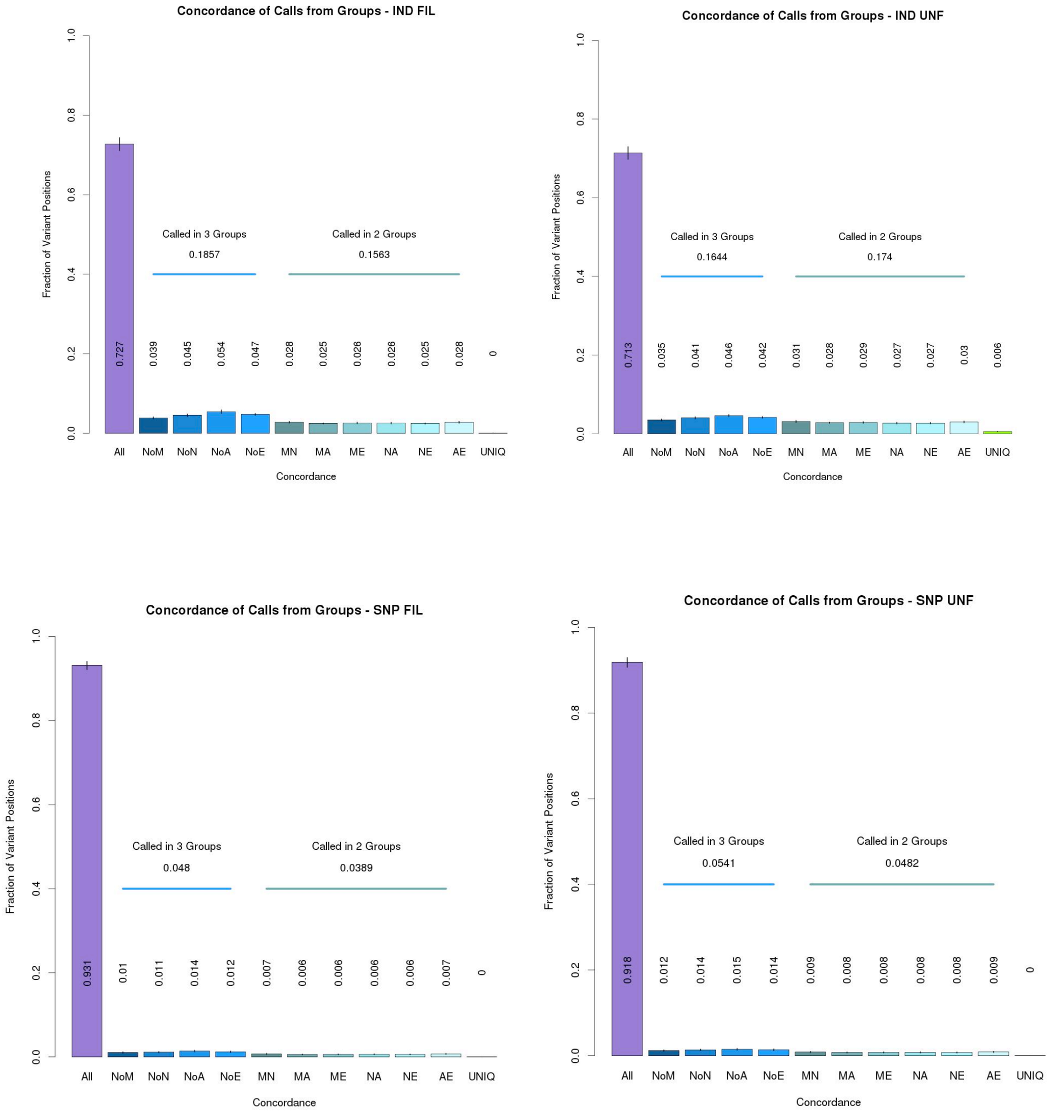

Supp. Figure 5:
Concordance of variant calls made on NA12878 amongst 4 ancestral test groups. Bars show mean proportion of concordant variant calls made on each of four chromosomes. 4 plots refer to concordance for filtered indels (top-left), unfiltered indels (top-right), filtered SNPs (bottom-left), and unfiltered SNPs (bottom-right)

SUPP FIGURE 6

A

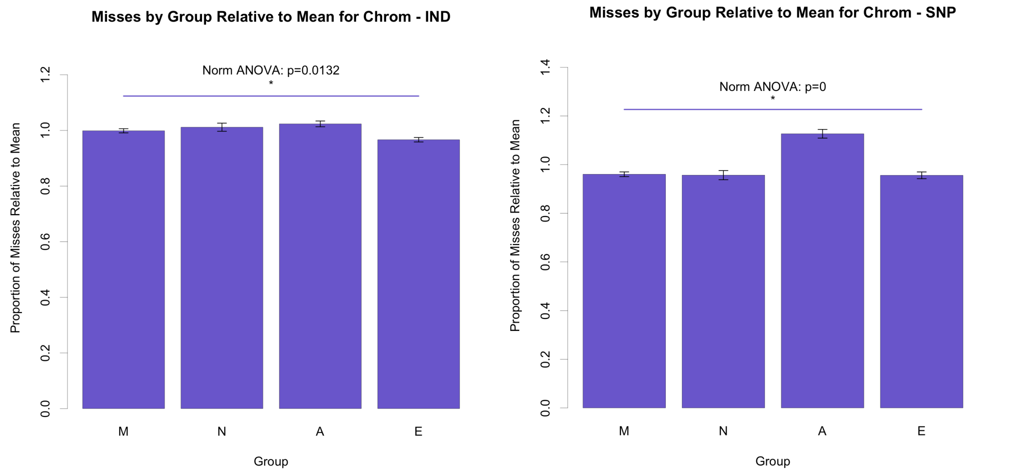

B

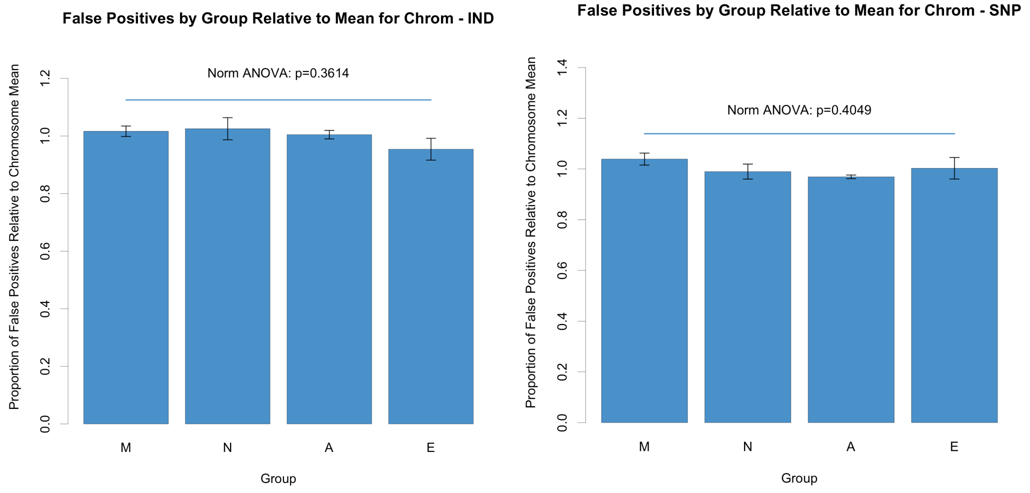

Supp. Figure 6:

(A) Mean (amongst 4 chromosomes) number of missed SNPs (left) and indels (right) for each ancestral test group

(B) Mean (amongst 4 chromosomes) number of false-positive SNPs (left) and indels (right) for each ancestral test group

SUPP FIGURE 7

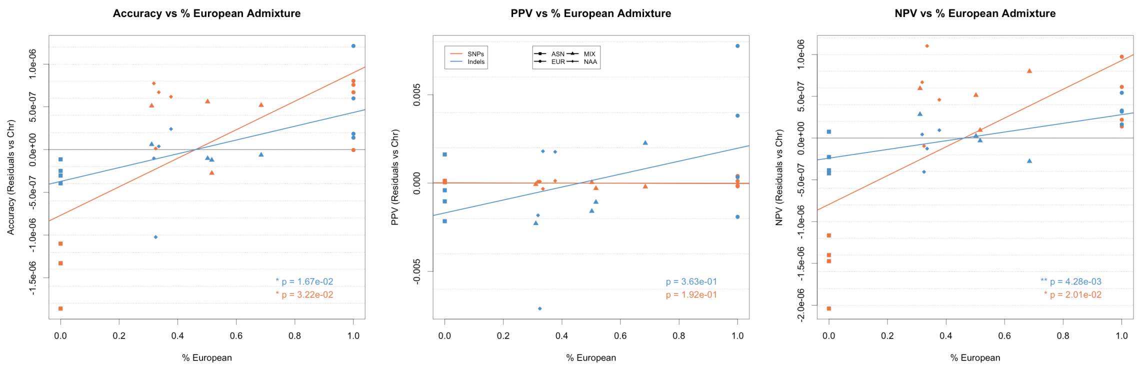

Supp. Figure 7:

Accuracy, positive predictive value (PPV), and negative predictive value (NPV), normalized by genomic region, versus fraction European admixture of test groups for variant calling.

SUPP FIGURE 8

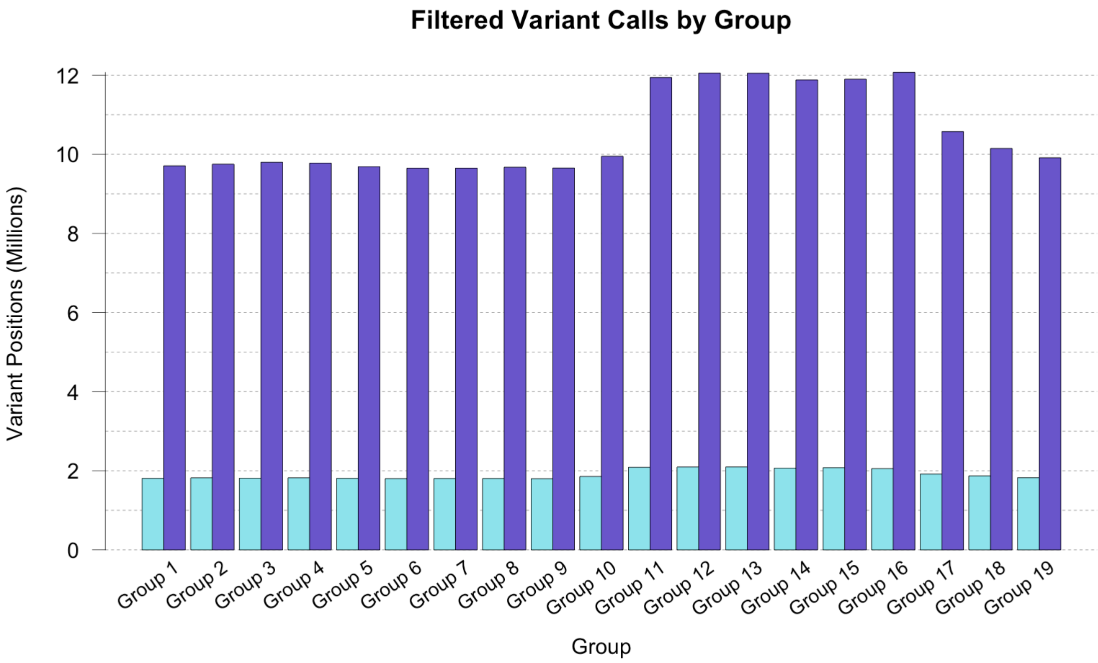

Supp. Figure 8:
Number of SNPs (purple) and indels (blue) called in each group after filtering.

SUPP FIGURE 9

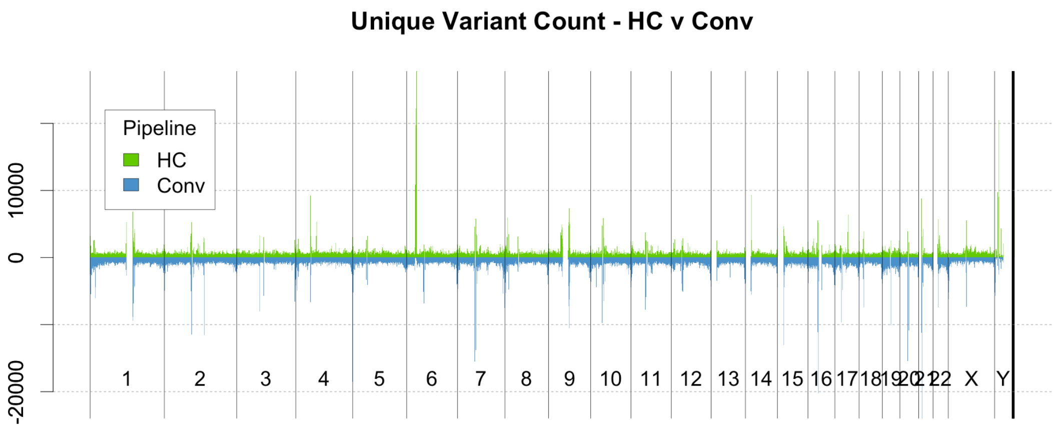

Supp. Figure 9:
Number of variant call positions (per 1MB) made exclusively by each pipeline (green=HaplotypeCaller; blue=Conventional)

SUPP FIGURE 10

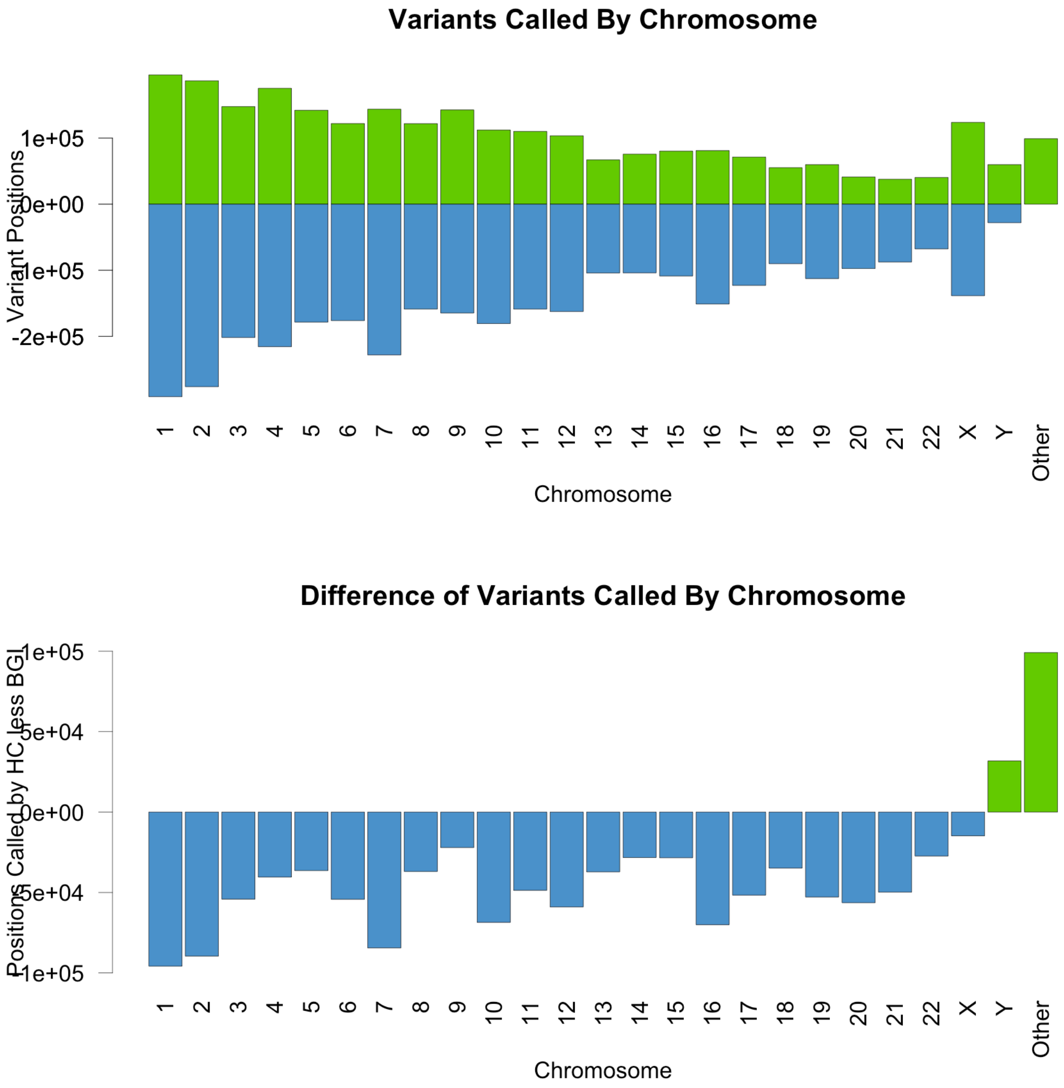

Supp. Figure 10:
Number of variants calls made exclusively by each pipeline per chromosome.
(excluding HLA region)
(green=HaplotypeCaller; blue=Conventional)

SUPP FIGURE 11

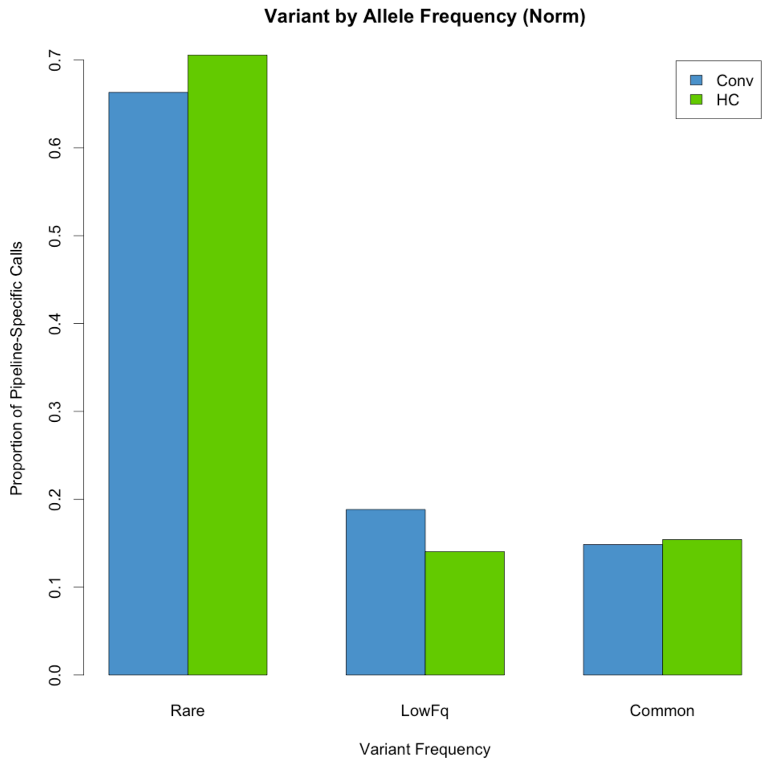

Supp. Figure 11:
Number of variant calls made exclusively by each pipeline, by minor allele frequency (Rare=MAF<1%; LowFq=1%<MAF<5%; Common=MAF>5%)

SUPP FIGURE 12

A

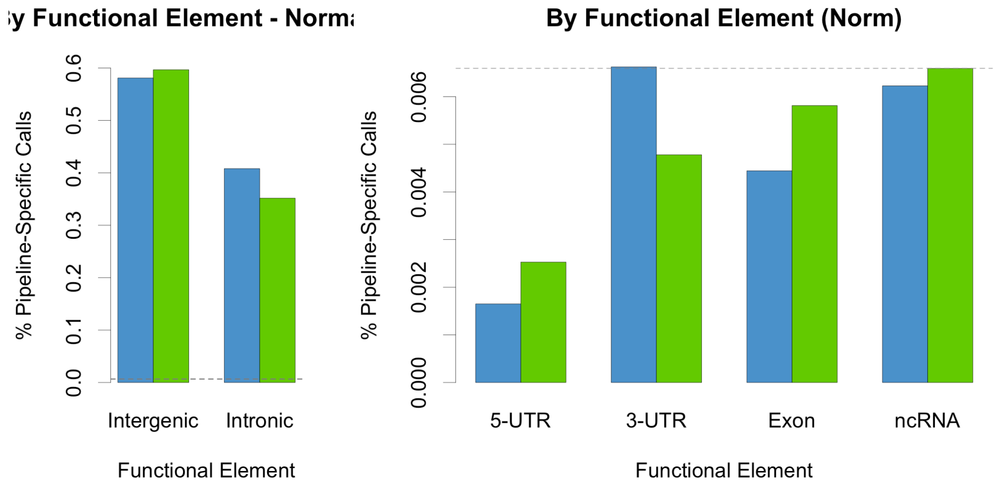

B

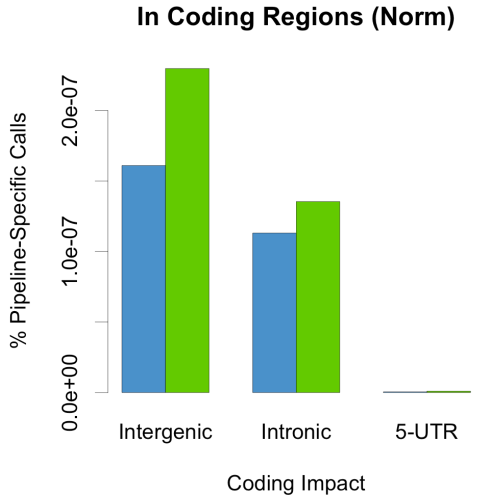

Supp. Figure 12:

(A) Proportion of variant calls made exclusively by each pipeline by location relative to functional element.

(B) Proportion of variant calls made exclusively by each pipeline by predicted coding impact.

SUPP TABLE 1

Step	Tool	Commands per Sample	Cores per Command	Commands per Node	Wall_Time per Sample (hr)	CPU_Time per Sample (hr)	SUs per Sample	MEM per Command (GB)	VMEM per Command (GB)	Output_File per Sample (GB)
FQ	NA	NA	NA	NA	NA	NA	NA	NA	NA	104.66+/-8.23
Map	BWA	4.83+/-0.54	11.47+/-3.97	1.39+/-4.03	12.4+/-3.12	96.48+/-9.33	133.5+/-28.13	6.81+/-0.56	7.55+/-0.73	415.14+/-30.14
Bam	Samtools	4.83+/-0.55	1.02+/-0.14	15.74+/-115.06	14.58+/-3.62	9.24+/-1.4	14.85+/-4.31	0.01+/-0	0.05+/-0.01	132.28+/-10.65
Merge	Samtools	1+/-0	1.02+/-0.33	15.71+/-47.78	8.04+/-1.02	7.09+/-0.6	8.28+/-4.89	0+/-0	0.05+/-0.01	132.11+/-10.16
Sort	Samtools	0.98+/-0.13	1.06+/-0.75	15.09+/-21.38	13.12+/-3.25	9.38+/-0.63	13.38+/-3.56	1.81+/-0.02	2.02+/-0.05	98.58+/-7.73
MarkDups	Picard	1+/-0	2.01+/-0.15	7.95+/-109.97	15.73+/-1.66	15.49+/-1.62	31.69+/-4.4	5.55+/-0.01	7.35+/-0.03	101.52+/-7.87
TrgtCrtr	GATK	1+/-0	2.02+/-0.16	7.93+/-103.12	8.89+/-0.57	8.64+/-0.55	17.99+/-2.22	2.34+/-0.16	7.42+/-0.29	0.05+/-0.01
IndlRlgnr	GATK	1+/-0.05	3.39+/-0.47	4.72+/-33.93	13+/-1.48	12.94+/-1.51	44.39+/-10.12	5.51+/-0.18	15.03+/-0.03	103.27+/-7.99
BsRecal	GATK	1+/-0.05	8.07+/-0.76	1.98+/-20.98	5.94+/-0.72	28.17+/-2.68	47.88+/-6.02	21.02+/-1.64	24.86+/-3.39	0.01+/-0
PrintReads	GATK	4.1+/-0.38	11.43+/-4.18	1.4+/-3.83	17.54+/-1.42	77.17+/-25.03	202.77+/-68.87	15.66+/-2.13	25.08+/-5.24	198.56+/-14.03
HaploCall	GATK	4	16	1	NA	NA	264.37+/-21.21	29.51+/-4.75	60.04+/-0.00	NA

Supp. Table 1

Step by Step Summary Statistics for Processing 437 Whole Genomes.
